# Association between clinically relevant antibiotic-resistant biliary colonization and liver-specific complications following perihilar cholangiocarcinoma resection

**DOI:** 10.1007/s00423-026-04157-5

**Published:** 2026-07-17

**Authors:** Faruk Koca, Hanan El Youzouri, Svenja Sliwinski, Konstantin Uttinger, Ursula Pession, Ekaterina Petrova, Dirk Walter, Michael Hogardt, Volkhard A. J. Kempf, Andreas A. Schnitzbauer, Armin Wiegering, Tamás Benkö

**Affiliations:** 1 Department of General, Visceral, Transplant and Thoracic Surgery, University Hospital, Goethe University Frankfurt, Frankfurt am Main, Germany; 2 Department of Gastroenterology, Hepatology, Endocrinology and Nutrition, University Hospital Frankfurt, Goethe University Frankfurt, Frankfurt am Main, Germany; 3 Institute for Medical Microbiology and Infection Control, University Hospital, Goethe University Frankfurt, Frankfurt am Main, Germany; 4 Department of Surgery, Knappschaft Kliniken University Hospital Bochum, Ruhr-University Bochum, Bochum, German Germany; 5 Department of General, Visceral, Transplant, and Thoracic Surgery, Goethe University Hospital Frankfurt, Theodor-Stern-Kai 7, 60590 Frankfurt, Germany

**Keywords:** Perihilar cholangiocarcinoma, Klatskin tumor, Biliary bacteria, Antibiotic-resistant bacteria, Bile leak

## Abstract

**Background:**

International guidelines recommend first-generation cephalosporins for preoperative antibiotic prophylaxis in patients undergoing resection for perihilar cholangiocarcinoma (pCCA). Knowledge on the resistance profile of biliary bacteria and its impact on liver-specific complications is limited. This study aimed to evaluate the biliary microbial spectrum, particularly focusing on the impact of resistant bacteria on liver-specific complications following pCCA resection.

**Methods:**

This is a retrospective, single-center, observational study. All patients with resected pCCA at the University Hospital Frankfurt from July 2005 to December 2022 were included. The microbial spectrum was analyzed using intraoperative bile swabs.

**Results:**

From 118 patients, 104 had an intraoperative bile swab taken. Microorganisms (bacteria, fungi) were detected in 89.4% of cases. 24% of the samples contained clinically relevant antibiotic-resistant bacteria, which were related with more severe postoperative bile leaks requiring relaparotomy (24% versus 8.8%, *p* = 0.055) and posthepatectomy liver failure (48% versus 25.3%, *p* = 0.031). 76.9% of the microbial isolates were resistant to in-house standard antibiotic prophylaxis with cefuroxime.

**Conclusion:**

Biliary colonization with clinically relevant antibiotic-resistant bacteria is associated with liver-specific complications.

**Supplementary information:**

The online version contains supplementary material available at 10.1007/s00423-026-04157-5.

## Introduction

Systemic therapy is only moderately effective for cholangiocarcinoma, so that surgery plays a crucial role in the treatment of patients. Postoperative morbidity about 65% [1] and mortality 14% [2] for perihilar cholangiocarcinoma (pCCA) surgery are among the highest for abdominal surgeries. Reported in-hospital mortality rates range from, depending on the extent of the resection performed [1].

The international guideline for perioperative management following liver resection recommends first-generation cephalosporins, such as cefazolin, for patients with unaffected bile ducts. Broader-spectrum agents, such as ceftriaxone or piperacillin-tazobactam, are only conditionally recommended for high-risk patients. Patients at higher risk for surgical site infections are defined as having one or more of the following risk factors: underlying liver disease, preoperative biliary drainage, or synchronous gastrointestinal resections [3]. However, not all patients with preoperative biliary drainage undergo culture testing/microbiological diagnosis.

Sugawara et al. demonstrated an association between multidrug-resistant bacteria and surgical site infections in patients undergoing major hepatectomy with extrahepatic bile duct resection [4]. However, they did not focus specifically on pCCA with liver-specific complications, especially bile leak. A previous study found high rates of concordance in the bacterial spectrum of the biliary tract and surgical site infections following hepatectomy and biliary reconstruction [5], but also did not focus on bile leak.

Broad-spectrum antibiotic therapy reduces overall morbidity after pancreatic head resection, especially in cases involving preoperative biliary drainage and bile duct contamination [6, 7]. However, the effectiveness of broad-spectrum anti-infective therapy for liver resections with preoperative biliary drainage similarly to pancreatic head resection remains unclear.

The goal of this study was to evaluate the resistance profile of biliary bacteria and its impact on liver-specific complications, especially in regard to bile leak.

## Materials and methods

This retrospective, single-center, observational study was approved by the local ethics committee of the Faculty of Medicine at the Goethe University Frankfurt, Germany (reference number: 2023 − 1607). All patients with pCCA, who underwent resection in curative intent at the University Hospital Frankfurt from July 2005 to December 2022, were included. Patients without intraoperative bile swab were excluded from the analysis. Data were collected retrospectively from the electronic patients’ records.

Demographic parameters (age at the time of surgery, sex), the presence of liver-specific conditions (primary sclerosing cholangitis, choledocholithiasis, steatohepatitis, fibrosis/cirrhosis, history of viral hepatitis), and tumor classification according to Bismuth-Corlette [8] were recorded. Surgical procedure was classified as follows: bile duct resection only, right hepatectomy (segments 5 to 8 ± 1), extended right hepatectomy (segments 4 to 8 ± 1), left hepatectomy (segments 2–4 ± 1), and extended left hepatectomy (segments 2–5 + 8 ± 1) [9]. Other parameters recorded were postoperative complications according the Clavien-Dindo classification [10], postoperative liver failure [11], bleeding [12] or bile leak [13] according to the International Study Group for Liver Surgery (ISGLS) classifications.

### Microbiological analysis

Swab tubes (e.g., Transystem sterile transport swab, suitable for aerobes and anaerobes, COPAN, Brescia, Italy) were used to collect a culture swab from bile duct after transsection of the bile duct for resection. The samples were analyzed by the Institute of Medical Microbiology and Infection Control to determine the bile duct microbial spectrum of patients with resected pCCA (fully certified since the year 2010 according to ISO 15189; certificate number D–ML–13102–01–00).

Cultures from patient specimens, bacterial species identification, and antibiotic susceptibility testing were performed using standard clinical microbiology methods. Antibiotic susceptibility testing was carried out. Vancomycin-resistant *Enterococci* (VRE) and carbapenemase-resistant Gram-negative bacteria were further analyzed by molecular identification techniques.

## Definitions

The following samples were defined as clinically relevant antibiotic-resistant bacteria (ARB): samples including Gram-negative bacteria, which are resistant to at least one third-generation cephalosporin or one of carbapenems, and vancomycin-resistant *Enterococci*. The mortality rate is higher for bloodstream infections caused by carbapenem-non-susceptible *Enterobacterales *[14] and vancomycin-resistant *Enterococci *[15]. The presence of Gram-negative bacteria resistant to third-generation cephalosporins poses a significant threat to public health [16]. Due to this background and the use of cephalosporins for single-dose antibiotic prophylaxis in-house, we included these bacteria in the ARB.

### Statistical analysis

The data were pseudonymized and analyzed using IBM (Armonk, NJ, USA) SPSS.

Statistics Subscription version 29.0.2.0 (20). The correlation between ARB and liver-specific complications, particularly severe bile leakage requiring a relaparotomy for resected pCCA, was analyzed.

Patients with ARB were compared with non-ARB patients. Interval-scaled secondary endpoints and other interval-scaled parameters were presented as medians with interquartile ranges (IQRs). For “severe bile leak”, both the total number in the respective group and the frequency of grade III according to ISGLS [13] was reported. We used Pearson’s chi-squared test and Fisher’s exact test to examine differences in categorical variables. The Mann–Whitney U test was applied for non-dependent variables. A *p* value of less than 0.05 was considered statistically significant.

## Results

Overall, 118 patients were included. Of these, 82 (69.5%) were male and 36 (30.5%) were female. The median age at the time of surgery was 67 years (IQR 14).

According to the Bismuth-Corlette classification there were five (4.2%) type I, 15 (12.7%) type II, 20 (16.9%) type IIIa, 29 (24.6%) type IIIb, and 47 (39.8%) type IV. Twelve patients (10.2%) underwent bile duct resection only, while the remaining patients underwent hepatectomy. Most of these patients underwent extended hepatectomy (63 patients, 53.4%). Table 1 presents a comprehensive overview of the descriptive statistics.

**Table 1:** Descriptive statistics

	*N*(%)/median (IQR)
Variables	*N* = 118
Age at the time of surgery (years)	67 (14)
Sex	
Male	82 (69.5)
Female	36 (30.5)
Bismuth-Corlette classification	
I	5 (4.2)
II	15 (12.7)
IIIa	20 (16.9)
IIIb	29 (24.6)
IV	47 (39.8)
Postoperative complications (CDC)	
0	12 (10.2)
1	25 (21.2)
2	14 (11.9)
3a	17 (14.4)
3b	20 (16.9)
4	7 (5.9)
90-day mortality	20 (16.9)
Posthepatectomy liver failure (ISGLS)	36 (30.5)
Grade I	21 (17.8)
Grade II	10 (8.5)
Grade III	5 (4.2)
Posthepatectomy hemorrhage (ISGLS)	41 (34.8)
Grade I	13 (11)
Grade II	13 (11)
Grade III	15 (12.7)
Biliary leak (ISGLS)	52 (44.1)
Grade I	22 (18.6)
Grade II	15 (12.7)
Grade III	15 (12.7)

*CDC *Clavien-Dindo classification, *ERCP* endoscopic retrograde cholangiopancreatography, *IQR* interquartile range, *ISGLS* International Study Group for Liver Surgery, *N* number

An intraoperative bile swab was performed on 104 patients (88.1%). Microorganisms were detected in 93 of the samples (89.4%). Of the samples analyzed, 70 (67.3%) were positive for Gram-positive bacteria, 55 (52.9%) for Gram-negative bacteria, 2 (1.9%) for anaerobic bacteria, and 31 (29.8%) for fungi. 93 of the patients (78.8%) received postoperative anti-microbial therapy based on laboratory values and clinical findings. 76.9% of the microbial isolates patients were resistant to cefuroxime, which was administered as a single-dose antibiotic prophylaxis before surgery. Table 2 summarizes the microbial findings and antimicrobial therapy. Supplementary Table 1 lists all of the samples containing microorganisms and their corresponding resistant antibiotics.

**Table 2:** Summary of microbial findings and antimicrobial therapy of resected perihilar cholangiocarcinoma. *N* (%)

Intraoperative bile swab	104
Microorganisms detected	93 (89.4)
ARB detected	25 (24)
Gram-positive bacteria	70 (67.3)
Gram-negative bacteria	55 (52.9)
Anaerobic bacteria	2 (1.9)
Fungi	31 (29.8)
Cefuroxime resistance	
None	22 (21.2)
Resistance	80 (76.9)
Postoperative anti-microbial therapy	
None	12 (10.2)
1–5 days	55 (52.4)
> 5 days	38 (36.2)
Missing	16 (13.6)
Antifungal therapy	11 (9.3)

*ARB* clinically relevant antibiotic-resistant bacteria

Risk factors for biliary colonization, particularly ARB colonization, were analyzed. No statistically significant risk factors were identified for microbial biliary colonization based on comorbidities or liver-specific conditions. Patients who underwent ERCP preoperatively had statistically significantly more positive bile swabs for microorganisms (93.7% versus 44.4%, *p* < 0.001). Similarly, preoperative bile duct stenting was a statistically significant risk factor for microbial biliary colonization (71% versus 36.4%; *p* = 0.030).

The detection of ARB was associated with lower levels of albumin and hemoglobin, as well as higher levels of C-reactive protein. However, the remaining laboratory parameters, including bilirubin, creatinine, white blood cells, CA 19 − 9, and CEA, did not significantly impact the detection of ARB. The presence of preoperative jaundice, and liver fibrosis or cirrhosis was associated with the detection of ARB colonization of the biliary tract. Risk factors for biliary colonization, including ARB, are shown in Table 3.

**Table 3:** Risk factors for biliary colonization, including multidrug-resistant microorganisms in resected perihilar cholangiocarcinoma

Variables	*N* = 104	bile swab		*p*	ARB		*p*
*N*(%)/median (IQR)		negative	positive		no	yes	
		11 (10.6)	93 (89.4)		79 (76)	25 (24)	
Age	69 (14)	70 (13)	68.5 (14)	0.386	67 (14)	70 (18)	0.400
Female sex	30 (28.8)	3 (27.3)	27 (29)	1	24 (30)	6 (24)	0.539
Preoperative conditions							
Cardiovascular disease	39 (37.5)	3 (27.3)	36 (38.7)	0.530	26 (33)	13 (52)	0.086
Pulmonary disease	19 (18.3)	1 (9.1)	18 (19.4)	0.684	15 (19)	4 (16)	0.736
PSC	4 (3.8)	0	4 (4.3)	1	4 (5)	0	0.251
Choledocholithiasis	10 (9.6)	2 (18.2)	8 (8.6)	0.285	7 (8.9)	3 (12)	0.643
Viral hepatitis	11 (10.6)	2 (18.2)	9 (9.7)	0.328	7 (8.9)	4 (16)	0.312
Fibrosis or cirrhosis	19 (18.3)	1 (9.1)	18 (19.4)	0.684	11 (13.9)	8 (32)	0.041
Jaundice	80 (76.9)	7 (63.6)	73 (78.5)	0.273	56 (70.8)	24 (96)	0.009
ERCP	95 (91.3)	6 (54.5)	89 (95.7)	< 0.001	72 (91.1)	23 (92)	0.894
Stenting	70 (67.3)	4 (36.4)	66 (71)	0.030	50 (62.3)	20 (80)	0.137
Laboratory parameters							
Bilirubin (mg/dl)	1.5 (4)	2.3 (4.2)	1.5 (4.6)	0.882	1.6 (4.6)	1 (3.8)	0.510
Creatinine (mg/dl)	0.8 (0.3)	0.8 (0.3)	0.8 (0.3)	0.530	0.8 (0.3)	0.9 (0.4)	0.749
Albumin (g/dl)	3.9 (0.8)	4.1 (0.9)	3.8 (0.7)	0.321	4 (0.7)	3.7 (0.8)	0.028
Hemoglobin (g/dl)	12.5 (2.1)	13 (3.4)	12.4 (2)	0.347	12.6 (2.2)	11.9 (1.9)	0.005
Leucocytes (/nl)	8.1 (4.2)	7.1 (2.7)	8.7 (4.3)	0.535	8.2 (4)	7.2 (5)	0.579
C-reactive protein (mg/dl)	1.3 (3.3)	0.4 (0.9)	1.6 (3.2)	0.629	1.3 (3.3)	2 (3.4)	0.026
CA 19 − 9 (U/ml)	132 (407)	49 (239)	162 (436)	0.216	186 (467)	34 (111)	0.632
CEA (µg/l)	2.5 (4)	2.2 (1.6)	3 (4.8)	0.761	2.4 (4.3)	3.6 (3.1)	0.342

*ERCP* endoscopic retrograde cholangiopancreatography, *IQR* interquartile range, *ARB* clinically relevant antibiotic-resistant bacteria, *PSC* Primary sclerosing cholangitis, *N* number

There was no statistically significant difference in the types of surgical procedures performed on patients with and without ARB biliary colonization. A total of 52 patients (41.1%) developed a bile leak. Of those patients, 15 (12.7%) had a grade III leak according to the ISGLS and underwent relaparotomy. Patients with ARB had a considerably higher rate of grade III bile leak requiring relaparotomy: 24% versus 8.9% (*p* = 0.055). It was also associated with more posthepatectomy liver failure (PHLF): 48 versus 25% (*p* = 0.031). Consequently, the rate of postoperative complications graded as 3B or higher according to the CDC was higher with ARB (60% versus 38%, *p* = 0.049). On the other hand, colonization with ARB was not significantly associated with hemorrhage or 90-day mortality. Table 4 provides a detailed overview of bile swab outcomes involving ARB.

**Table 4:** Analysis of biliary colonization with clinically relevant antibiotic-resistant bacteria in resected perihilar cholangiocarcinoma

				*p* value
		ARB		
*N* = 104		no	yes	
*N*(%)		79 (76)	25 (24)	
Surgical procedure				
Bile duct resection only	10 (9.6)	7 (8.9)	3 (12)	0.643
Right hepatectomy	18 (17.3)	14 (17.7)	4 (16)	0.843
Extended right hepatectomy	32 (30.8)	27 (34.2)	5 (20)	0.181
Left hepatectomy	19 (18.3)	13 (16.5)	6 (24)	0.395
Extended left hepatectomy	25 (24)	18 (22.8)	7 (28)	0.595
Orifices of hepaticojejunostomy				0.539
1	40 (38.5)	32 (40.5)	8 (32)	
2	40 (38.5)	28 (35.4)	12 (48)	
3	10 (9.6)	10 (12.7)	0	
4	13 (12.5)	8 (10.1)	5 (20)	
Bile leak grade III (ISGLS)	13 (12.5)	7 (8.9)	6 (24)	0.055
PHLF	32 (30.8)	20 (25.3)	12 (48)	0.031
Hemorrhage	38 (36.5)	28 (35.4)	10 (40)	0.427
Complications ≥3b to CDC	45 (43.3)	30 (38)	15 (60)	0.049
90-day mortality	19 (21.2)	14 (17.7)	5 (20)	0.502

*ARB* clinically relevant antibiotic-resistant bacteria, *CDC *Clavien-Dindo classification, *ERCP* endoscopic retrograde cholangiopancreatography, *ISGLS *International Study Group for Liver Surgery, *PHLF* poshepatectomy liver failure

## Discussion

Over the course of 17 years, 118 patients in our cohort with pCCA underwent curative resection. 104 patients underwent an intraoperative bile swab. Microorganisms were detected in 89.4% of cases. Of these samples, 76.9% included microorganisms resistance to cefuroxime, a single-dose antibiotic prophylaxis administered in-house. 24% belonged to the ARB group, which showed more severe postoperative bile leak grade III according to ISGLS (*p* = 0.055) and posthepatectomy liver failure (*p* = 0.031).

Preoperative cholangitis and bacterial bile colonization increase the risk of surgical site infections and mortality following resection of pCCA [17]. Cammann et al. found that antibiotic-resistant bacteria in the bile duct are associated with increased morbidity and mortality. This is especially apparent with ciprofloxacin-resistant bacteria, which have been linked to a higher risk of surgical complications in patients undergoing resection for extrahepatic cholangiocarcinoma [18]. Unlike the recent study, this study did not focus solely on pCCA, but also distal cholangiocarcinoma. It also did not thoroughly evaluate the association between liver-specific complications, particularly bile leak. Instead, this study examined ciprofloxacin-resistant bacteria in patients with postoperative complications requiring surgery. However, cefazolin, not ciprofloxacin, is the recommended antibiotic for single-dose prophylaxis.

Sugarawa et al. found that a positive bile culture for multidrug-resistant pathogens prior to surgery was a significant risk factor for postoperative infections in a multivariate analysis. However, they did not find an association between multidrug-resistant pathogens and bile leak. They compared grades B and C together but not grade C alone, as was done in the current study [4]. Similarly to liver resection with biliary colonization, the risk of major infectious morbidity following pancreatic resection for cancer is significantly associated with antimicrobial resistance [19]. On the other hand, selective decontamination of the digestive tract may negatively impact postoperative outcomes, particularly following pancreatic resection [20]. Bile leak is more common in pCCA and is associated with major complications and surgical site infections [21]. The current study revealed a higher association between ARB and severe bile leak requiring relaparotomy (24% versus 8.9%). However, according to Fisher’s exact test, this association was not statistically significant (*p* = 0.055). Relaparotomy was indicated if the patient was septic and did not respond to antibiotics or if a bile leak involved higher volumes. When hepaticojejunostomy insufficiency was occured, the procedure was re-performed with biliary drainage and peritoneal lavage. There was no association between ARB and 90-day mortality. The mortality rate was 16.9%, which is comparable to the rate of 17.9% reported in previous single-center studies, such as that of Bednarsch et al. [22]. The causes of death were PHLF in four patients, bleeding in three patients, coecal volvulus in one patient, mesenteric ischemia in one patient, cardiogenic shock in two patients, and unknown in seven patients. Only two patients died from a bile leak. It is possible that managing bile leak through relaparotomy could avoid the septic consequences that could result in death.

The risk factors for ARB could be explained with previous antibiotic use. Hirayama et al. demonstrated that higher resistance rates were associated with piperacillin/tazobactam and ceftriaxone. The detection of carbapenemase-producing, carbapenem-non-susceptible *Enterobacterales* was associated with the use of piperacillin/tazobactam. Non-carbapenemase-producing, carbapenem-non-susceptible *Enterobacterales* were associated with the overall antimicrobial use density, as well as with piperacillin/tazobactam and ceftriaxone use [14]. As our institution is a tertiary referral university hospital, many patients are initially diagnosed at secondary care hospitals, including undergoing ERCP. Consequently, information on preoperative antimicrobial therapy and its role in ARB is frequently unavailable. Preoperative ERCP with stenting is not the standard institutional procedure for diagnosing pCCA. It is only recommended for patients with severe cholangitis and hyperbilirubinemia.

78.8% of our cohort received postoperative anti-microbial therapy based on laboratory values and clinical findings. However, in most cases, this therapy was initiated only after receiving the results of microbiological analyses of intraoperative bile swabs, which take at least two to three days. If antimicrobial therapy is started too late, it may not be effective enough to prevent infection-related suture insufficiency. Since many patients require antimicrobial therapy based on clinical decision-making, we consider empiric perioperative antibiotic therapy. It could be administered preoperatively with the appropriate antibiotics for pCCA. This should be considered because pCCA has high rates of infectious morbidity and mortality.

Due to the issue of bile colonization, the international guideline recommendation of single-dose antibiotic prophylaxis for pCCA resection is not feasible. We observed 76.9% resistance to cefuroxime, similar to the 25% preoperative rate reported by Bednarsch et al. [22]. Yet, the international guidelines recommend first-generation cephalosporins [3].

Bile swabs during ERCP are not standard practice for gastroenterologists. Therefore, microbiological analyses from ERCPs are rare. On the other hand, microbial colonization may occur a few days after an ERCP. For this reason, we believe the practice of administering a single dose of antibiotics as prophylaxis with cefalozin for pCCA resection should change if bile colonization is considered. 

### Limitations

This is a retrospective, single-center study. Due to pronounced multidrug resistance, it is not possible to recommend an ideal single-dose antibiotic regimen. There is also insufficient evidence to support the benefits of empirical perioperative therapy. Additionally, previous antibiotic exposure is a major potential determinant of ARB colonization. However, this is 17-year, single-center, retrospective study, so information on previous antibiotic exposure is lacking. The limited sample size of 25 patients with ARB restricts the scope of the analysis.

## Conclusion

Biliary colonization with ARB is associated with liver-specific complications, especially PHLF after pCCA resection.

## Supplementary material

Below is the link to the electronic supplementary material.

Supplementary File 1(DOCX 36.2 KB)

## Data Availability

No datasets were generated or analysed during the current study.

## References

[1] Gerhards MF, van Gulik TM, de Wit LT, Obertop H, Gouma DJ (2000) Evaluation of morbidity and mortality after resection for hilar cholangiocarcinoma–a single center experience. Surgery 127(4):395–404. 10.1067/msy.2000.104250 10776430 10.1067/msy.2000.104250

[2] Olthof PB, Erdmann JI, Alikhanov R et al (2024) Higher Postoperative Mortality and Inferior Survival After Right-Sided Liver Resection for Perihilar Cholangiocarcinoma: Left-Sided Resection is Preferred When Possible. Ann Surg Oncol 31(7):4405–4412. 10.1245/s10434-024-15115-0 38472674 10.1245/s10434-024-15115-0 PMC11164810

[3] Maier E, Stättner S, Carrion-Alvarez L et a (2025) The E-AHPBA - ESSO - Innsbruck consensus recommendations on peri- and postoperative management following liver resection. HPB (Oxford) S1365-182X(25):01671-5. Published online. 10.1016/j.hpb.2025.10.008 10.1016/j.hpb.2025.10.008 41238460

[4] Sugawara G, Yokoyama Y, Ebata T et al (2018) Preoperative biliary colonization/infection caused by multidrug-resistant (MDR) pathogens in patients undergoing major hepatectomy with extrahepatic bile duct resection. Surgery 163(5):1106–1113. 10.1016/j.surg.2017.12.031 29398033 10.1016/j.surg.2017.12.031

[5] Makino K, Ishii T, Yoh T et al (2022) The usefulness of preoperative bile cultures for hepatectomy with biliary reconstruction. Heliyon 8(12):e12226. 10.1016/j.heliyon.2022.e12226 36568677 10.1016/j.heliyon.2022.e12226 PMC9768314

[6] Giannone F, Lagarrigue C, Ligurgo O et al (2025) Adaptation of antibiotics and antifungal strategy to preoperative biliary drainage to improve postoperative outcomes after pancreatic head resection. World J Surg 49(1):270–282. 10.1002/wjs.12446 39681975 10.1002/wjs.12446 PMC11711118

[7] D’Angelica MI, Ellis RJ, Liu JB et al (2023) Piperacillin-Tazobactam Compared With Cefoxitin as Antimicrobial Prophylaxis for Pancreatoduodenectomy: A Randomized Clinical Trial. JAMA 329(18):1579–1588. 10.1001/jama.2023.5728 37078771 10.1001/jama.2023.5728 PMC10119777

[8] Bismuth H, Nakache R, Diamond T (1992) Management Strategies in Resection for Hilar Cholangiocarcinoma. Ann Surg 215(1):31–38. 10.1097/00000658-199201000-00005 1309988 10.1097/00000658-199201000-00005 PMC1242367

[9] Strasberg SM (2005) Nomenclature of hepatic anatomy and resections: a review of the Brisbane 2000 system. J Hepatobiliary Pancreat Surg 12(5):351–355. 10.1007/s00534-005-0999-7 16258801 10.1007/s00534-005-0999-7

[10] Dindo D, Demartines N, Clavien PA (2004) Classification of Surgical Complications: A New Proposal With Evaluation in a Cohort of 6336 Patients and Results of a Survey. Ann Surg 240(2):205–213. 10.1097/01.sla.0000133083.54934.ae 15273542 10.1097/01.sla.0000133083.54934.ae PMC1360123

[11] Rahbari NN, Garden OJ, Padbury R et al (2011) Posthepatectomy liver failure: A definition and grading by the International Study Group of Liver Surgery (ISGLS). Surgery 149(5):713–724. 10.1016/j.surg.2010.10.001 21236455 10.1016/j.surg.2010.10.001

[12] Rahbari NN, Garden OJ, Padbury R et al (2011) Post-hepatectomy haemorrhage: a definition and grading by the International Study Group of Liver Surgery (ISGLS). HPB 13(8):528–535. 10.1111/j.1477-2574.2011.00319.x 21762295 10.1111/j.1477-2574.2011.00319.x PMC3163274

[13] Brooke-Smith M, Figueras J, Ullah S et al (2015) Prospective evaluation of the International Study Group for Liver Surgery definition of bile leak after a liver resection and the role of routine operative drainage: an international multicentre study. HPB 17(1):46–51. 10.1111/hpb.12322 25059275 10.1111/hpb.12322 PMC4266440

[14] Hirayama S, Otani M, Murakami H et al (2026) Association between antimicrobial use and the detection rates of carbapenem-resistant Enterobacterales: long-term surveillance results from a single centre. Infect Prev Pract 8(1):100500. 10.1016/j.infpip.2025.100500 41586323 10.1016/j.infpip.2025.100500 PMC12830325

[15] Hsu CC, Jung CJ, Yang JL et al (2026) Clinical outcomes and molecular epidemiology of vancomycin-resistant Enterococcus faecium bloodstream infection: The importance of appropriate therapy and ST17 predominance. J Infect Public Health 19(7):103274. 10.1016/j.jiph.2026.103274 42214909 10.1016/j.jiph.2026.103274

[16] Tajuelo A, Gato E, Moya C et al (2026) KpFhaB/FhaC is a virulence-associated TPS system of the globally-disseminated Klebsiella pneumoniae ST15 high-risk clone. Front Cell Infect Microbiol 16:1787622. 10.3389/fcimb.2026.1787622 42318020 10.3389/fcimb.2026.1787622 PMC13272123

[17] Sakata J, Shirai Y, Tsuchiya Y, Wakai T, Nomura T, Hatakeyama K (2009) Preoperative cholangitis independently increases in-hospital mortality after combined major hepatic and bile duct resection for hilar cholangiocarcinoma. Langenbecks Arch Surg 394(6):1065–1072. 10.1007/s00423-009-0464-1 19169703 10.1007/s00423-009-0464-1

[18] Cammann S, Karabulut S, DeTemple DE et al (2022) Antibiotic-Resistant Bacteria Colonizing the Bile Duct Are Associated with Increased Morbidity and Mortality after Resection of Extrahepatic Cholangiocarcinoma. Surg Infect (Larchmt) 23(3):270–279. 10.1089/sur.2021.117 35172114 10.1089/sur.2021.117

[19] Gianotti L, Tamini N, Gavazzi F et al (2017) Consequences of Increases in Antibiotic Resistance Pattern on Outcome of Pancreatic Resection for Cancer. J Gastrointest Surg 21(10):1650–1657. 10.1007/s11605-017-3483-1 28681215 10.1007/s11605-017-3483-1

[20] Mibelli N, Oehme F, Radulova-Mauersberger O et al (2024) Bacterial shift and resistance pattern in pancreatic head resections after selective decontamination of the digestive tract - a propensity score-matched analysis. J Gastrointest Surg 28(11):1844–1852. 10.1016/j.gassur.2024.08.030 39241947 10.1016/j.gassur.2024.08.030

[21] Vienet J, Labgaa I, Duran R et al (2025) Incidence and risk factors of biliary leaks after partial hepatectomy within an enhanced recovery perioperative pathway: a single-center retrospective cohort study. Langenbecks Arch Surg 410(1):104. 10.1007/s00423-025-03677-w 40131479 10.1007/s00423-025-03677-w PMC11937051

[22] Bednarsch J, Czigany Z, Heij LR et al (2021) Bacterial bile duct colonization in perihilar cholangiocarcinoma and its clinical significance. Sci Rep 11(1):2926. 10.1038/s41598-021-82378-y 33536484 10.1038/s41598-021-82378-y PMC7858613

