# Supplementary material for: Association between clinically relevant antibiotic-resistant biliary colonization and liver-specific complications following perihilar cholangiocarcinoma resection
Source: Langenbecks Arch Surg. 2026 Jul 17;411(1):195. doi: 10.1007/s00423-026-04157-5 (PMC13379436; doi:10.1007/s00423-026-04157-5)
Supplement: Supplementary file 1 — (DOCX 36.2 KB) [file 423_2026_4157_MOESM1_ESM.docx]

| Supplementary Table 1 Microorganisms and drug resistance profiles isolated from an intraoperative bile swab in perhilar cholangiocarcina resections. | | | |
| --- | --- | --- | --- |
| Sample number | ARB | Microorganisms | Resistance profile |
| 1 | + | *Klebsiella pneumoniae* (ESBL-R) | Amoxicillin/clavulanic acid R, piperacillin/tazobactam R, cefuroxime R, cefotaxime R, gentamicin HLR, levofloxacin R, ciprofloxacin R |
|  |  | *Citrobacter braaki* | Piperacillin/tazobactam I |
|  |  | *Aeromonas sobria* | Ampicillin R, amoxicillin/clavulanic acid R, piperacillin/tazobactam R, cefuroxime R |
|  |  | *Candida albicans* |  |
| 2 | + | *Stenotrophomonas maltophilia* |  |
|  |  | *Enterococcus faecium* |  |
|  |  | *Candida albicans* |  |
| 3 | + | *Escherichia coli* (ESBL-R) | Ampicillin R, amoxicillin/clavulanic acid R, piperacillin/tazobactam I, cefuroxime R, cefotaxime R, cotrimoxazole R, levofloxacin R, ciprofloxacin R |
|  |  | *Klebsiella pneumoniae* | Amoxicillin/clavulanic acid R, piperacillin/tazobactam R, cefuroxime R, cefotaxime R, gentamicin HLR, levofloxacin R, ciprofloxacin R |
| 4 | + | *Enterococcus faecium* | Vancomycin R |
|  |  | *Candida albicans* |  |
| 5 | + | *Escherichia coli* (ESBL-R) | Ampicillin R, amoxicillin/clavulanic acid R, piperacillin/tazobactam R, cefuroxime R, cefotaxime R, cotrimoxazole R, |
|  |  | *Enterococcus faecalis* | Rifampicin R |
|  |  | *Candida albicans* |  |
| 6 | + | *Stenotrophomonas maltophilia* |  |
| 7 | + | *Enterococcus faecium* | Vancomycin R |
|  |  | *Candida albicans* |  |
| 8 | + | *Klebsiella pneumoniae* (ESBL-R) | Amoxicillin/clavulanic acid R, cefuroxime R, cefotaxime R, cotrimoxazole R, levofloxacin R, ciprofloxacin R, tobramycin R, fosfomycin R |
|  |  | *Candida albicans* |  |
| 9 | + | *Enterococcus gallinarum* | Vancomycin R |
| 10 | + | *Enterococcus faecium* | Vancomycin R |
|  |  | *Enterococcus faecalis* |  |
| 11 | + | *Enterococcus faecium* | Vancomycin R |
|  |  | *Candida glabrata / albicans* |  |
| 12 | + | *Escherichia coli* |  |
|  |  | *Enterococcus faecium* | Gentamicin HLR |
|  |  | *Enterococcus casseliflavus* | Vancomycin R |
|  |  | *Candida glabrata / C. dubliniensis* |  |
| 13 | + | *Stenotrophomonas maltophilia* |  |
|  |  | *Escherichia coli* | Gentamicin HLR |
|  |  | *Enterococcus faecalis* |  |
|  |  | *Enterococcus faecium* |  |
| 14 | + | *Enterobacter cloacae* | Cefotaxime R, ceftriaxone R, cefepime R, tigecyclin R |
|  |  | *Enterobacter cloacae* |  |
|  |  | *Enterococcus faecalis* |  |
| 15 |  | *Enterobacter cloacae* |  |
|  |  | *Enterococcus faecium* | Tetracyclin R, rifampicin R |
|  |  | *Bacteroides species* |  |
|  |  | *Candida albicans* |  |
| 16 | + | *Enterococcus faecalis* |  |
|  |  | *Klebsiella oxytoca* |  |
|  |  | *Morganella morganii* | Piperacillin/tazobactam R, cefotaxime R, fosfomycin R |
|  |  | *Candida albicans* |  |
| 17 |  | *Enterococcus faecium* |  |
|  |  | *Enterococcus faecalis* |  |
|  |  | *Candida albicans* |  |
| 18 |  | Coagulase-negative *Staphylococcus* | Penicillin G R, oxacillin R, cefazolin R, cefuroxime R |
|  |  | *Candida albicans* |  |
| 19 |  | *Escherichia coli* |  |
|  |  | *Enterobacter cloacae* |  |
|  |  | *Enterococcus faecalis* | Gentamicin HLR |
| 20 | + | *Pseudomonas aeruginosa* | Piperacillin/tazobactam I, ceftriaxon R, ceftazidime I, cefepime I, imipenem R, meropenem R, levofloxacin R, ciprofloxacin R, fosfomycin R |
|  |  | *Enterococcus faecalis* | Gentamicin HLR, tetracyclin R, amikacin R, rifampicin R |
| 21 |  | *Enterobacter cloacae* |  |
|  |  | *Enterococcus faecalis* | Ampicillin R, imipenem R, gentamicin HLR |
|  |  | *Lactococcus lactis* |  |
| 22 |  | *Klebsiella oxytoca* |  |
|  |  | *Enterococcus faecium* |  |
| 23 |  | *Staphylococcus aureus* |  |
| 24 |  | *Morganella morganii* |  |
| 25 |  | *Enterococcus faecalis* |  |
|  |  | *Enterococus faecium* | Gentamicin HLR |
| 26 |  | *Hafnia alvei* |  |
|  |  | *Enterococcus faecalis* | Gentamicin HLR |
| 27 |  | *Escherichia coli* |  |
|  |  | *Enterococcus faecalis* | Gentamicin HLR |
|  |  | *Candida albicans* |  |
| 28 |  | *Escherichia coli* | Piperacillin/tazobactam R, gentamicin HLR, cotrimoxazole R, levofloxacin R |
|  |  | *Enterococcus faecalis* | Gentamicin HLR |
|  |  | *Enterococcus faecium* |  |
| 29 |  | *Streptococci* | Gentamicin HLR, tetracyclin R, levofloxacin R, ciprofloxacin R, tobramycin R |
|  |  | *Candida albicans* |  |
| 30 |  | *Enterobacter cloacae* | Fosfomycin R |
|  |  | *Enterococcus faecalis* |  |
| 31 |  | *Candida parapsilosis* |  |
| 32 |  | *Enterococcus faecium* |  |
|  |  | *Enterobacter amnigenus* |  |
| 33 |  | *Enterococcus faecalis* |  |
|  |  | *Escherichia coli* |  |
|  |  | *Candida albicans* |  |
| 34 |  | *Enterococcus faecalis* |  |
| 35 |  | *Proteus mirabilis* |  |
|  |  | *Klebsiella pneumoniae* | Amoxicillin/clavulanic acid R |
| 36 |  | *Klebsiella pneumoniae* | Cefuroxime R, tigecyclin R |
| 37 |  | *Enterococcus faecalis* |  |
|  |  | *Candida albicans* |  |
| 38 |  | *Candida albicans* |  |
| 39 |  | *Klebsiella pneumoniae* |  |
|  |  | *Hafnia alvei* |  |
|  |  | *Enterococcus faecalis* | Tetracyclin R |
| 40 |  | *Staphylococcus aureus* |  |
| 41 |  | *Klebsiella pneumoniae* |  |
|  |  | Enterococcus faecalis |  |
| 42 |  | *Enterobacter cloacae* |  |
|  |  | *Enterococcus faecalis* | Tetracyclin R |
| 43 |  | *Enterococcus faecalis* |  |
|  |  | *Bacillus species* |  |
| 44 |  | *Enterococcus faecalis* | Tetracyclin R |
| 45 |  | *Enterobacter cloacae* |  |
|  |  | *Citrobacter braakii* |  |
| 46 | + | *Enterobacter cloacae* | Piperacillin/tazobactam I, cefotaxime R, ceftriaxone R, tigecyclin R |
| 47 |  | *Enterococcus faecalis* | Gentamicin HLR |
|  |  | *Enterococcus faecium* |  |
| 48 |  | *Pantoea spp.* |  |
| 49 |  | *Enterococcus faecium* |  |
| 50 |  | *Enterococcus faecalis* | Tetracyclin R |
| 51 |  | *Escherichia coli* |  |
|  |  | *Enterobacter cloacae* | Fosfomycin R |
|  |  | *Morganella morganii* | Fosfomycin R |
| 52 |  | *Enterococcus faecalis* |  |
| 53 |  | *Enterococcus faecium* | Tetracyclin R |
| 54 |  | *Coagulase-negative staphylococci* | Penicillin G R, levofloxacin R, erythromycin R |
| 55 |  | *Enterobacter cloacae* | Tigecyclin I, fosfomycin R |
| 56 |  | *Klebsiella oxytoca* |  |
|  |  | *Enterobacter cloacae* |  |
|  |  | *Enterococcus faecalis* |  |
|  |  | *Bacteroides species* |  |
| 57 |  | *Citrobacter braakii* | Piperacillin/tazobactam R |
| 58 |  | *Enterococcus faecalis* |  |
|  |  | *Staphylococcus epidermidis* | Penicillin G R, oxacillin R, cefazolin R, cefuroxime R, levofloxacin R, fosfomycin R |
| 59 |  | *Enterococcus faecalis* |  |
| 60 | + | *Enterococcus faecalis* | Rifampicin R |
|  |  | Enterococcus casseliflavus | Vancomycin R |
|  |  | *Citrobacter freundii* |  |
|  |  | *Klebsiella oxytoca* |  |
| 61 |  | *Enterobacter aerogenes* |  |
|  |  | *Candida tropicalis / albicans* |  |
| 62 |  | *Candida albicans* |  |
| 63 |  | *Enterococcus faecium* | Tetracyclin R |
|  |  | *Candida utilis* |  |
| 64 |  | *Enterococcus faecium* | Gentamicin HLR, tetracyclin R |
|  |  | *Enterococcus faecalis* | Gentamicin HLR, tetracyclin R |
|  |  | *Candida albicans* |  |
| 65 |  | *Klebsiella oxytoca* | Fosfomycin R |
|  |  | *Lactobacillus rhamnosus* |  |
| 66 |  | *Klebsiella pneumoniae* | Amoxicillin/clavulanic acid I, tigecycline R |
| 67 | + | *Enterobacter cloacae* | Piperacillin/tazobactam R, cefotaxime R, tigecycline R |
|  |  | *Enterococcus faecalis* |  |
|  |  | *Candida albicans* | Fluconazole R |
| 68 |  | *Coagulase-negative staphylococci* |  |
| 69 |  | *Enterococcus faecalis* | Tetracyclin R |
|  |  | *Enterococcus faecium* |  |
| 70 |  | *Klebsiella pneumoniae* |  |
| 71 |  | *Streptococcus gordonii* | Erythromycin R |
| 72 | + | *Citrobacter werkmanii* | Piperacillin/tazobactam R, cefotaxime R |
|  |  | *Raoultella (Klebsiella) planticola* |  |
| 73 |  | *Citrobacter freundii* |  |
|  |  | *Enterococcus faecalis* |  |
|  |  | *Candida albicans, Candida spp.* (Non *- albicans)* |  |
| 74 |  | *Enterococcus faecalis* |  |
| 75 |  | *Enterococcus faecium* | Gentamicin HLR |
|  |  | *Candida albicans* |  |
| 76 |  | *Enterobacter cloacae complex* | Fosfomycin R |
|  |  | *Actinomyces odontolyticus* | Penicillin G I |
| 77 |  | *Enterococcus faecium* |  |
|  |  | *Enterococcus faecalis* |  |
| 78 |  | *Raoultella (Klebsiella) ornithinolytica* |  |
|  |  | *Enterobacter cloacae complex* | Imipenem I |
|  |  | *Enterococcus faecium* | Gentamicin HLR |
| 79 |  | *Pseudomonas aeruginosa* | Ciprofloxacin R |
|  |  | *Enterococcus faecalis* |  |
|  |  | *Candida albicans, Candida spp. (Non - albicans)* |  |
| 80 | + | *Enterobacter cloacae complex* | Piperacillin/tazobactam R, cefotaxime R, fosfomycin R |
|  |  | *Enterococcus faecalis* |  |
| 81 |  | *Klebsiella pneumoniae* | Levofloxacin I, ciprofloxacin R |
|  |  | *Enterococcus faecalis* |  |
|  |  | *Candida albicans* |  |
| 82 |  | *Candida dubliniensis, Candida kefyr (pseudotropicalis),* |  |
|  |  | *Rothia mucilaginosa* | Penicillin G R, cotrimoxazole R |
|  |  | *Granulicatella adiacens* | Penicillin G I, cefotaxime I |
| 83 |  | *Enterococcus faecalis* |  |
| 84 |  | *Enterobacter cloacae complex* | Fosfomycin R |
|  |  | *Escherichia coli* |  |
|  |  | *Enterococcus faecalis* | Gentamicin HLR |
| 85 | + | *Enterobacter cloacae complex* | Piperacillin/tazobactam R, cefotaxime R |
|  |  | *Enterococcus faecalis* |  |
| 86 |  | *Klebsiella oxytoca* | Ampicillin/sulbactam R, piperacillin/tazobactam R, cefuroxime R |
|  |  | *Enterococcus faecalis* |  |
| 87 |  | *Enterococcus faecalis* |  |
| 88 |  | *Staphylococcus epidermidis* | Oxacillin R, cefazolin R, cefuroxime R, levofloxacin R |
|  |  | *Streptococcus mitis/oralis* |  |
| 89 |  | *Streptococcus mitis/oralis* | Clindamycin R |
| 90 |  | *Escherichia coli* | Ampicillin R, ampicillin/sulbactam R |
|  |  | *Klebsiella pneumoniae* | Ampicillin/sulbactam R |
|  |  | *Enterococcus faecalis* |  |
| 91 | + | *Enterococcus faecium* |  |
|  |  | *Acinetobacter pittii* |  |
|  |  | *Candida albicans* |  |
|  |  | *Candida parapsilosis* | Caspofungin I, micafungin I |
| 92 | + | *Enterobacter cloacae complex* | Piperacillin/tazobactam R, cefotaxime R, fosfomycin R |
|  |  | *Enterococcus faecalis* |  |
| 93 | + | *Enterobacter cloacae* |  |
|  |  | *Pseudomonas aeruginosa* |  |
|  |  | *Candida albicans* |  |
| ARB- samples including clinically relevant antibiotic-resistant bacteria, ESBL-R- extended-spectrum β-lactamase resistant, I- intermediate, R- resistant | | | |
